# Physicochemical Characteristics and Flavor Quality Analysis of Fermented Jerky from Yanbian Beef Cattle

**DOI:** 10.3390/foods14020300

**Published:** 2025-01-17

**Authors:** Xiao Yang, Changlei Liu, Qi Wang, Enying Cui, Hongjie Piao, Yuping Wen, Guanhao Li, Qing Jin

**Affiliations:** 1 College of Agriculture, Yanbian University, Yanji 133002, China; y2891472@gmail.com (X.Y.); 0000008083@ybu.edu.cn (C.L.); wangqi1234561216@163.com (Q.W.); 0000008592@ybu.edu.cn (H.P.); 0000008711@ybu.edu.cn (Y.W.); 2 School of Public Health, Jilin University, Changchun 130012, China; cey0103@163.com; 3 Key Innovation Laboratory for Deep and Intensive Processing of Yanbian High Quality Beef, Ministry of Agriculture and Rural Affairs, Yanji 133002, China

**Keywords:** Yanbian yellow cattle, fermented beef jerky, physicochemical properties, flavor quality

## Abstract

Beef jerky is a traditional meat product. It is uses beef as the main raw material, and is processed through multiple procedures such as curing, maturing, drying, sterilization, and packaging. However, changes in raw materials, curing solution, the choice of fermenter, and fermentation conditions affect the quality and flavor of beef jerky. Therefore, we investigated the effects of inoculation with *Pentosaccharomyces schizococcus* and *Staphylococcus veal*, both pre- and post-fermentation, on the physicochemical characteristics and flavor quality of Yanbian beef jerky. Key parameters, including pH, water activity, fundamental nutrients, and color, were measured, while qualitative and flavor characteristics were assessed using a texture meter, an electronic nose, and an electronic tongue. The results indicated that samples inoculated with the composite fermenter exhibited significant increases in ash content, hardness, total free amino acid concentration, and levels of specific flavor-enhancing amino acids compared to unfermented jerky (*p* < 0.05). In contrast, moisture content, pH, and water activity were significantly reduced (*p* < 0.05). Three fatty acids—heptadecenoic acid, trans-oleic acid, and arachidonic acid—were identified for the first time in the fermented beef jerky. Furthermore, during the fermentation process, saturated fatty acid content was reduced by 21.88%, while polyunsaturated fatty acid content increased by 29.58% (*p* < 0.05).

## 1. Introduction

Beef jerky is a traditional meat product. It is primarily composed of beef, and is processed through several steps, including curing, maturing, drying, sterilization, and packaging [1]. This process not only effectively preserves the rich nutrients of beef, but also significantly enhances the product’s portability. The flavor characteristics of beef jerky are greatly influenced by the quality of the raw beef and the production process [2]. Yanbian yellow beef, one of the top five yellow cattle breeds in China, is characterized by tender, juicy, flavorful, and nutrient-rich meat. It is rich in proteins, minerals, unsaturated fatty acids, and amino acids beneficial to human health, with particularly high levels of oleic acid compared to other beef breeds [3]. However, limited research has focused on the application of fermented meat products. The production process plays a crucial role in the flavor of beef jerky, with the fermentation process being especially important. Fermentation imparts a unique flavor to beef jerky while also extending shelf life and enhancing food safety [4]. Compared with natural fermentation, the artificial addition of fermentation agents allows for more effective control over the quality of beef jerky [5]. During the fermentation process, hydrolytic enzymes such as protease and lipase promote the catabolism of proteins and fats in the meat, which not only improves the digestibility and absorption of the products but also plays a crucial role in enhancing the product’s flavor [6]. In addition, lactic acid, a sugar metabolite, effectively lowers the pH of beef jerky, thereby inhibiting the growth of spoilage organisms and pathogenic bacteria [7].

Currently, microbial fermenters commonly used in meat fermentation include *lactic acid bacteria*, *staphylococci*, *micrococci*, *yeasts*, and *molds*. *Lactic acid bacteria* primarily produce lactic acid by metabolizing carbohydrates, along with small amounts of organic acids such as acetic, formic, and succinic acids, which inhibit the growth of spoilage bacteria while contributing a distinctive flavor to fermented meat products [8]. *Staphylococci* are closely associated with the development of color during meat processing [9]. To date, most studies have focused on fermenting meat using either a single strain or a combination of two strains. Some scholars studied the fermentation of beef jerky from bacon by using *Staphylococcus xylosus* P_2_ as a fermenter, and the results showed that beef jerky inoculated with this fermenter was significantly better than that of the uninoculated group in terms of color, flavor, texture, etc. [10]. Researchers also produced fermented beef jerky by inoculating with *Pediococcus acidilactici* BP2 (BP), and found that the pH of the inoculated beef jerky was reduced, and the color, texture, and flavor were significantly improved. This was potentially due to the decomposition of organic acids by the fermenter, resulting in a reduction in pH and an improvement in flavor, as well as accelerated protein hydrolysis, which improved the texture [11]. In addition, fermented beef jerky was produced via inoculation with *Staphylococcus xyloglucosus* and *Lactobacillus sakeus*. It was found that the hardness and chewing were significantly reduced in the group inoculated with the fermenter (*p* < 0.05), but that the redness was significantly higher than in the group without inoculation (*p* < 0.05), the redness value and free amino acid content increased significantly, and the proportion of fresh flavor amino acids was the highest. Additionally, the free fatty acids were mainly palmitic acid, stearic acid, oleic acid, and linoleic acid, of which oleic acid accounted for the largest proportion [5].

Therefore, in this study, Yanbian yellow beef tendon meat was used as a raw material, and fermentation was initiated by inoculation with *Pentosaccharomyces schizococcus* and *Staphylococcus calvus*. The aim was to investigate changes in the physicochemical properties and flavor quality of Yanbian yellow beef jerky after fermentation in order to provide a scientific foundation and reference for the development of Yanbian yellow beef meat products.

## 2. Materials and Methods

### 2.1. Materials and Reagents

*Pentosaccharomyces cerevisiae* (KCCM 11902) was purchased from the Korean Culture Center of Microorganisms (Seoul, Korea). *Staphylococcus veal* (CICC 10850) was purchased from the China Industrial Microbial Strain Conservation and Management Center (Beijing, China).

Yanbian yellow beef tendon meat, granulated sugar, sesame oil, soy sauce, pepper, and sugar dilution were purchased from Yanji Department Store Supermarket; anhydrous ethanol, sodium chloride, and glycerol were purchased from the Sinopharm Chemical Reagent Co. (Shanghai, China).

### 2.2. Yanbian Yellow Beef Jerky Production

The leg muscles of Yanbian yellow cattle carcasses were selected as raw materials, with fat and other non-essential tissues removed. Then, they were cut into smaller pieces. They were cleaned and thoroughly mixed with seasoning, including onion, sugar, sesame oil, soy sauce, and sugar dilution. The meat was marinated for four hours and then inoculated with *Pentosaccharomyces pentosaceus* and *Staphylococcus carnosus* to ensure uniformity. After ensuring a homogeneous mixture, the mixture was placed in a constant-temperature incubator to undergo the fermentation process. The fermented beef was boiled and subsequently dried at 75 °C for three hours to produce fermented beef jerky samples from Yanbian yellow cattle. The preparation process for the unfermented beef jerky followed the same steps; however, after marination, the meat was placed directly into the constant-temperature incubator, without the inoculation of the compound fermenter, to complete the process.

### 2.3. Determination of General Composition of Beef Jerky

A fully automated Kjeldahl nitrogen tester (K9860, Hanon, Jinan, China) was employed for the precise determination of the crude protein content. A Soxhlet extractor (SZF-06C, Zjtpyq, Shanghai, China) was utilized for fat content determination. An infrared moisture meter (CSY-H5A, Csy17, Shenzhen, China) was subsequently employed to measure moisture content [12]. Ash content was determined with reference to Sam et al. [13].

### 2.4. Determination of Physicochemical Properties of Beef Jerky

The pH was measured using a pH meter (PHS-3C, INESA, Shanghai, China), while nitrite and nitrate concentrations were quantified using the naphthylenediamine hydrochloride method [14]. Biogenic amines were quantified using spectrophotometry (U-3900, Hitachi, Tokyo, Japan) [15,16]. The hardness, viscosity, cohesion, elasticity, and chewiness of beef jerky were analyzed using texture profiling [17]. The color of the samples was measured using a colorimeter (WSC-S, Shanghaijingke, Shanghai, China), and the data obtained were expressed as brightness (L-value), redness (a-value), and yellowness (b-value) [18]. The moisture activity values in beef jerky were measured using a moisture activity meter (LabMASTER, Novasina, Switzerland) [19]. Finally, thiobarbituric acid reactive substances (TBARS) in the samples were quantified using a spectrophotometer (U-3900, Hitachi, Tokyo, Japan) [20].

The fatty acid composition was determined using a gas-phase mass spectrometer (QP2010, Shimadzu, Kyoto, China), equipped with a mass spectrometric capillary column featuring a poly (dicyanopropylsiloxane) strong polar stationary phase (100 m × 0.25 mm, 0.2 μm), and a GC-FID detector [21]. The inlet temperature was set at 250 °C, while the detector temperature was set at 260 °C. The programmed temperature increase was as follows: the initial temperature of 100 °C was maintained for 13 min; it was then increased to 180 °C at a rate of 10 °C/min and this was held for 6 min; subsequently, it was raised to 200 °C at a rate of 1 °C/min and maintained for 20 min; finally, it was increased to 230 °C at a rate of 4 °C/min and held for 10.5 min. The carrier gas used was nitrogen. 

The amino acids in beef jerky were hydrolyzed using hydrochloric acid (6 M), separated, and reacted with ninhydrin. Subsequently, the composition and content of the amino acids were quantified using a fully automated amino acid analyzer (L-8900, Hitachi, Japan) [21]. The conditions for the analysis were as follows: we used a chromatographic column containing sulfonic acid cationic resin, with proline content detected at 440 nm and 16 free amino acids, including leucine, isoleucine, and alanine, detected at 570 nm.

### 2.5. Determination of Flavor Quality of Beef Jerky

A 3 g sample was accurately weighed and placed in a sealed sample bottle. This was equilibrated for 15 min and subsequently analyzed using an electronic nose sensor (PEN3, AIRSENSE, Schwerin, Germany) [22].

A 9 g sample of churned material was weighed and mixed with 90 mL of distilled water for 10-fold dilution. This solution was then homogenized and centrifuged at 10,000 r/min for 10 min at 4 °C. The mixture was subsequently filtered through three layers of gauze, and the resulting filtrate was transferred to a beaker designated for the electronic tongue. The filtrate was then tested for acidity, bitterness, astringency, and saltiness using the Electronic Tongue Sensing System (SA204B, Insent, Atsugi, Japan), allowing for the determination of the five basic tastes: sweet, sour, salty, bitter, and umami [22].

### 2.6. Microbial Community Detection During Beef Fermentation Process

Nucleic acids were extracted from beef following the fermentation process described in Section 2.2 using the OMEGA Soil DNA Kit (Omegabiotek, Guangzhou, China). The molecular size of the extracted nucleic acids was assessed using 0.8% agarose gel electrophoresis, and the DNA concentration was quantified using a UV spectrophotometer (LUX, Thermo Scientific, Waltham, MA, USA). We selected primers that were specific to the V3-V4 region of bacterial 16S rDNA, namely, 338F (5′-barcode + ACTCCTACGGGGAGGCAGCA-3′) and 806R (5′-GGACTACHVGGGTWTCTAAT-3′). The thermal cycling conditions included pre-denaturation at 98 °C for 30 s, denaturation at 98 °C for 15 s, annealing at 50 °C for 10 s, and extension at 72 °C for 30 s. This was followed by 25–27 cycles, and a final extension at 72 °C for 300 s. The qualified products were analyzed using the Illumina NovaSeq platform from Parsons Brinckerhoff Biotechnology Co., Ltd. (Illumina, San Diego, CA, USA), employing a 2 × 250 bp sequencing approach.

### 2.7. Data Processing and Statistical Analysis

The data obtained were analyzed and plotted using GraphPad Prism 10 software. One-way ANOVA was conducted using SPSS 27.0 software, and comparisons were performed using the Waller–Duncan method. Results were deemed statistically significant when the *p*-value was less than 0.05. The results of the experimental measurement indicators were reported to two decimal places and expressed as the mean ± standard error of the mean.

## 3. Results and Discussion

### 3.1. Physicochemical Properties of Beef Jerky

The physicochemical properties of beef jerky are presented in Table 1. The study results indicated that, compared to the unfermented group, the fermented group exhibited a significant increase (*p* < 0.05) in ash content, while crude protein content, moisture content, pH, and water activity were significantly lower (*p* < 0.05). Conversely, fat content, nitrite residue, histamine content, and TBARS values did not differ significantly between the two groups. These findings align with those of Zhang et al. [23], who investigated the physicochemical characteristics of yeast-inoculated fermented bacon and reported a significant decrease in crude protein content, moisture content, pH, and water activity compared to the natural fermentation group. The increase in ash content could be attributed to the enhanced bioavailability of minerals during fermentation. In contrast, the decrease in protein content potentially resulted from the degradation of protein into low-molecular-weight peptides and free amino acids by proteases produced by microorganisms and endogenous enzymes present in the beef during fermentation [23]. The reduction in water content could be attributed to the fermentation group’s pH being close to the isoelectric point of muscle proteins, resulting in the denaturation of these proteins and the contraction of muscle bundles. This process led to a loss of water within the protein network and between myogenic fibers, ultimately decreasing water retention. The reduction in water activity could be attributed to the transformation of sugars and other substances into organic acids, such as lactic acid, by *Pentosaccharomyces schizophilus* during fermentation. This transformation resulted in a decrease in pH, which could have lead to protein denaturation or degradation, subsequently weakening the ability to retain water [24]. The decrease in pH resulted from the strong acid-producing capacity of *Pentosaccharomyces schizococcus*, which decomposed carbohydrates in beef to produce lactic acid and other byproducts. Additionally, fat content, nitrite residue, histamine content, and TBARS values did not differ significantly between the two groups.

The physicochemical properties of beef jerky are presented in Table 1. The results indicated that the hardness of the fermented group was significantly greater than that of the unfermented group (*p* < 0.05). In contrast, no significant differences were observed between the two groups concerning viscosity, cohesion, elasticity, chewiness, and L-, a-, and b-values (*p* > 0.05). These findings align with the results reported by Shikha et al. [25], who fermented beef jerky with *Lactobacillus* both prior to and following fermentation. The observed increase in hardness was attributable to the denaturation of proteins and the reduction in moisture content during fermentation, factors which both significantly impacted the texture of the beef jerky [26].

### 3.2. Free Amino Acid Composition of Beef Jerky

Amino acids are pivotal determinants of the flavor of meat products, wherein the amino acids and peptides generated through protein hydrolysis can impart various flavor characteristics, including freshness, sweetness, and bitterness. The levels of free amino acids in beef jerky and their specific concentrations are presented in Table 2. The results indicated that the fermented group exhibited a significant increase in the concentrations of fresh, sweet, and bitter amino acids compared to the unfermented beef jerky group. Additionally, the concentrations of essential amino acids, non-essential amino acids, and total amino acids increased by 10.83%, 15.31%, and 13.55%, respectively. This change was consistent with the findings of Zhang et al. [27], who investigated the alterations in the free amino acid content of sour meat through complex fermentation with *Lactobacillus* and *Pentosaccharomyces schizococcus*. In their study, the concentrations of essential amino acids, non-essential amino acids, and total amino acids in the complex fermentation group were higher than those in the natural fermentation group by 36.93%, 33.84%, and 35.49%, respectively. The protease activity exhibited by the inoculated *Staphylococcus vealensis* likely promotes protein hydrolysis, subsequently resulting in the production of additional amino acids. Moreover, certain amino acids function as precursors to flavor compounds, which can further be converted into volatile flavor compounds, thereby enhancing the overall flavor profile of the product [28].

### 3.3. Free Fatty Acid Composition of Beef Jerky

Free fatty acids, as flavor precursors, can decompose into smaller molecular compounds, such as aldehydes and ketones, leading to a pleasant and distinctive flavor [29]. The compositions and concentrations of free fatty acids in beef jerky are presented in Table 3. The results indicate that a total of 13 fatty acids were detected in the fermented group, which represented an increase of 3 compared to the unfermented group, specifically heptadecenoic acid, trans-oleic acid, and the essential fatty acid arachidonic acid. Furthermore, the concentration of monounsaturated fatty acids in the fermented group increased by 6.02%, while the concentration of polyunsaturated fatty acids rose by 29.58%. This observation aligns with the trends in fatty acid changes observed during the fermentation process of golden pomfret fish, as investigated by Wang et al. [30]. The content of unsaturated fatty acids increased following fermentation, particularly docosahexaenoic acid, eicosapentaenoic acid, and docosapentaenoic acid. This change potentially resulted from the proliferation of inoculated *Pentosaccharomyces schizococcus* and *Staphylococcus carnosus* during fermentation, which promoted the conversion and breakdown of saturated fatty acids [31].

### 3.4. Beef Jerky Flavor Quality

**Figure 1:**
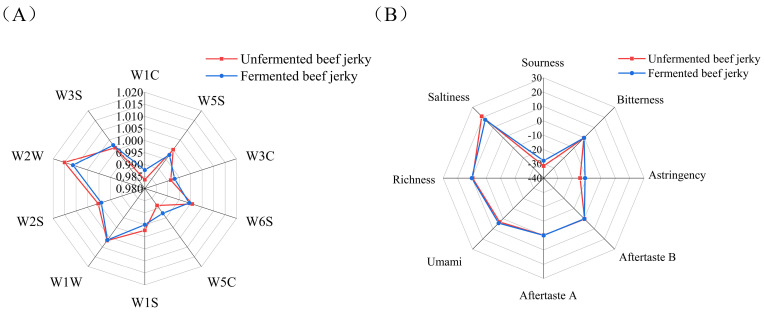
Flavor quality of beef jerky. (**A**) Electronic nose flavor analysis of beef jerky. (**B**) Electronic tongue flavor analysis of beef jerky). Note: FJ denotes the group of fermented beef jerky from the Yanbian yellow cattle inoculated with a compound fermenter; WFJ denotes the group of fermented beef jerky from Yanbian yellow cattle not inoculated with a compound fermenter.

The flavor profiles of the two sample groups were examined using the electronic nose technique, with the results presented in Figure 1A. The results indicated that the response values of beef jerky in the fermented group were significantly enhanced for the W5S (sensitive to nitrogen oxides) and W1W (sensitive to sulfur compounds) sensors compared to those of the unfermented group; the response values from the other sensors remained relatively unchanged. This change was attributed to the addition of *Staphylococcus* to the fermented beef jerky, which affected the degradation of proteins and fats, consequently altering the type and content of flavoring substances, and thereby enabling the sensors to capture distinct odor signals [32].

Eight taste characteristics of beef jerky—sourness, bitterness, astringency, bitter aftertaste, astringent aftertaste, freshness, richness/freshness aftertaste, and saltiness—were analyzed using the electronic tongue technique, and the results are presented in Figure 1B. In comparison to the unfermented group, the fermented group exhibited increased astringency, sourness, and freshness, along with decreased saltiness. The increase in sourness is closely associated with the fermentation process. Furthermore, freshness, an essential factor influencing product quality, enhances the richness and complexity of flavor perception. The enhancement of freshness may result from the release of flavor-presenting amino acids, facilitated by the fermentation process [33]. The aforementioned sensors effectively distinguish unfermented beef jerky from its fermented counterpart.

### 3.5. Microbial Community Analysis During Beef Fermentation Process

#### 3.5.1. Analysis of Beef Alpha Diversity

**Figure 2:**
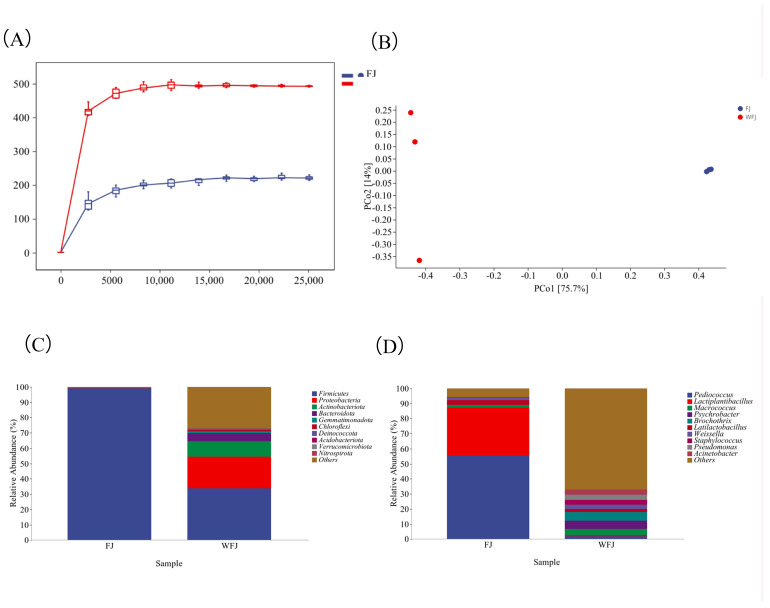
The analysis of the microbial community structure of beef jerky. (**A**): Beef bacterial sparsity curve; (**B**): PCA of the microbial community structure of beef; (**C**): composition of bacterial communities in beef at the phylum level; (**D**): composition of bacterial communities in beef at the genus level. Note: FJ denotes the group of fermented beef jerky from Yanbian yellow cattle inoculated with compound fermenter; WFJ denotes the group of fermented beef jerky from Yanbian yellow cattle not inoculated with compound fermenter.

The V3–V4 variable region of the 16S rDNA gene in beef was sequenced and analyzed before and after fermentation. A total of 251,455 high-quality sequences were obtained from unfermented beef, averaging 83,818 per sample, whereas 209,343 high-quality sequences were obtained from fermented beef, averaging 69,781 per sample. These sequences were subsequently categorized into operational taxonomic units (OTUs). Figure 2A illustrates that the dilution curves of all samples leveled off, indicating that the current sequencing volume was sufficiently large to accurately reflect the bacterial community structure of the beef samples. Using OTU annotation, 15 bacterial phyla, 24 bacterial orders, and 104 bacterial genera were identified in unfermented beef, whereas 7 bacterial phyla, 9 bacterial orders, and 37 bacterial genera were identified in fermented beef.

Based on the OTUs, the Shannon and Chao1 indices were calculated for the bacterial communities of unfermented and fermented beef, respectively, to characterize the alpha diversity of the samples. The results presented in Table 4 indicate that the Chao1 value and Shannon index of unfermented beef were significantly higher than those of fermented beef, suggesting a notable difference in alpha diversity between the two. This finding aligns with the study by Sha et al. [34], where fermentation significantly reduced the α-diversity of fermented beef jerky, as observed in reductions in the Chao1, Shannon, and Simpson indices of α-diversity. Following inoculation with the fermenter, the dominant bacteria likely inhibited the growth and reproduction of other microorganisms by competing for nutrients and ecological niches.

#### 3.5.2. Beef Microbial Community Structure

The bacterial community structures of unfermented and fermented beef were analyzed in detail at the taxonomic levels of the phylum and genus. The phyla that ranked among the top ten in mean relative abundance were classified as dominant phyla, and the genera that ranked among the top ten were classified as dominant genera. Figure 2C indicates that the dominant phyla in unfermented beef primarily include *Firmicutes*, *Proteobacteria*, and *Actinobacteria*. The abundance of the dominant genera, listed in descending order, was as follows: *Macrococcus*, *Psychrobacter*, *Brochothrix*, *Latilactobacillus*, *Weissella*, *Staphylococcus*, *Pseudomonas*, and *Acinetobacter*.

Figure 2D shows that the *phylum Firmicutes* (also referred to as thick-walled bacteria) was overwhelmingly dominant in fermented beef, accounting for 99.15% of the total abundance. At the genus level, the genera with higher abundance included *Schizococcus*, *Lactiplantibacillus*, *Latilactobacillus*, *Megacoccus*, *Weissella*, and *Staphylococcus*. These results suggest that the fermentation process reduces microbial diversity and may inhibit the proliferation of undesirable bacteria. This finding differs from the study by Sha et al. [34] regarding bacterial community changes at the genus level in fermented beef jerky, which can be attributed to variations in dominant genera resulting from differences in fermenter inoculation. However, the changes during fermentation were similar, likely because genera such as *Lactobacillus* possess protease and lipase activities, enhancing the flavor profile of beef jerky.

#### 3.5.3. Beef Beta Diversity Analysis

Beta diversity analysis revealed the variability in microbial community structures among different beef samples. The bacterial community structure of beef samples was analyzed using principal component analysis, as illustrated in Figure 2B. The bacterial community structure of the two beef samples exhibited contributions of 75.7% from the first principal component and 14.0% from the second principal component, resulting in a cumulative contribution of approximately 89.7%. Unfermented and fermented beef samples were positioned farther apart on the PCA plot, indicating significant differences in their bacterial community structures.

## 4. Conclusions

Yanbian yellow beef jerky fermented with the addition of *Pentosaccharomyces schizococcus* and *Staphylococcus vealensis* demonstrated improved texture and color, reduced pH and water activity, and the inhibition of lipid overoxidation, nitrite residues, and histamine accumulation. The proteins and lipids present in the beef jerky were decomposed and oxidized into amino acids, fatty acids, and other compounds, enhancing texture and contributing to flavor development. In conclusion, compared with unfermented beef jerky, the texture and color of fermented beef jerky were substantially enhanced, with physicochemical properties and flavor profiles notably improving. However, the system for producing fermented beef jerky from Yanbian Huang beef remains relatively complex. Further studies are required to elucidate the fermentation mechanisms in different parts of Yanbian Huang beef responsible for producing its distinctive flavor.

## Figures and Tables

**Table 1:** Physicochemical properties of beef jerky.

Indicator	Unfermented Beef Jerky	Fermented Beef Jerky
Protein content (%)	60.60 ± 0.35 ^a^	58.84 ± 0.42 ^b^
Fat content (%)	3.97 ± 0.21 ^a^	3.85 ± 0.80 ^a^
Moisture content (%)	19.96 ± 0.40 ^a^	17.52 ± 0.41 ^b^
Ash content (%)	3.21 ± 0.09 ^b^	3.89 ± 0.08 ^a^
pH value	5.77 ± 0.03 ^a^	4.96 ± 0.05 ^b^
Aw value	0.78 ± 0.00 ^a^	0.76 ± 0.01 ^b^
Nitrite residue/(mg/kg)	0.01 ± 0.00 ^a^	0.01 ± 0.00 ^a^
Histamine/(mg/100 g)	5.47 ± 0.03 ^a^	5.49 ± 0.02 ^a^
TBRAS value/(mg/100 g)	0.59 ± 0.06 ^a^	0.63 ± 0.01 ^a^
Hardness/N	166.22 ± 9.89 ^b^	201.27 ± 12.76 ^a^
Viscosity/mJ	0.22 ± 0.03 ^a^	0.21 ± 0.05 ^a^
Cohesion	0.35 ± 0.03 ^a^	0.30 ± 0.02 ^a^
Elasticity/mm	6.61 ± 0.55 ^a^	6.97 ± 0.05 ^a^
Chewability/mJ	380.66 ± 19.81 ^a^	423.45 ± 46.71 ^a^
L*	46.68 ± 0.78 ^a^	47.83 ± 0.45 ^a^
a*	2.58 ± 0.39 ^a^	3.13 ± 0.23 ^a^
b*	5.57 ± 0.84 ^a^	6.79 ± 0.46 ^a^

Note: the different letter indicates a significant difference (*p* < 0.05).

**Table 2:** Free amino acid composition of beef jerky.

Amino Acid Classification	Amino Acid Name	(mg/kg)	
		Unfermented Beef Jerky	Fermented Beef Jerky
Fresh amino acids	Aspartic acid (Asp)	14.131 ± 0.536 ^b^	15.980 ± 0.462 ^a^
	Glutamine (Glu)	25.496 ± 0.179 ^b^	30.627 ± 0.963 ^a^
	Total	39.628 ± 0.716 ^b^	46.607 ± 1.425 ^a^
Sweet amino acids	Alanine (Ala)	9.082 ± 0.359 ^b^	10.299 ± 0.285 ^a^
	Glycine (Gly)	6.508 ± 0.030 ^b^	7.637 ± 0.199 ^a^
	Serine (Ser)	6.378 ± 0.250 ^b^	7.211 ± 0.208 ^a^
	Threonine (Thr)	7.321 ± 0.467 ^a^	8.012 ± 0.254 ^a^
	Total	29.289 ± 1.105 ^b^	33.159 ± 0.946 ^a^
Bitter amino acids	Phenylalanine (Pre)	6.327 ± 0.239 ^b^	7.162 ± 0.158 ^a^
	Methionine (Met)	4.064 ± 0.164 ^a^	4.456 ± 0.172 ^a^
	Leucine (Leu)	12.020 ± 0.474 ^b^	13.539 ± 0.392 ^a^
	Isoleucine (Iso)	5.448 ± 0.224 ^a^	5.956 ± 0.195 ^a^
	Valine (Val)	5.583 ± 0.228 ^a^	6.086 ± 0.194 ^a^
	Cysteine (Gys)	0.692 ± 0.014 ^b^	0.867 ± 0.017 ^a^
	Histidine (His)	4.767 ± 0.191 ^b^	5.490 ± 0.044 ^a^
	Arginine (Arg)	10.371 ± 0.386 ^b^	11.535 ± 0.354 ^a^
	Total	49.273 ± 1.919 ^b^	55.091 ± 1.528 ^a^
Odorless amino acids	Lysine (Lys)	13.189 ± 0.551 ^a^	14.587 ± 0.513 ^a^
	Tyrosine (Tyr)	5.488 ± 0.227 ^a^	5.963 ± 0.239 ^a^
	Total	18.677 ± 0.778 ^a^	20.550 ±0.753 ^a^
Essential amino acid (EAA)		53.952 ± 2.946 ^a^	59.798 ± 1.879 ^a^
Non-essential amino acid (NEAA)		82.914 ± 2.173 ^b^	95.609 ± 2.722 ^a^
Total amino acid (TAA)		136.866 ± 4.519 ^b^	155.408 ± 4.651 ^a^

Note: the different letter indicates a significant difference (*p* < 0.05).

**Table 3:** Free fatty acid composition of beef jerky.

Fatty Acids		Content (g/100 g)	
		Unfermented Beef Jerky	Fermented Beef Jerky
Saturated fatty acid (SFA)	Lauric acid C12:0	0.002 ± 0.001 ^a^	0.001 ± 0.000 ^b^
	Myristic acid C14:0	0.045 ± 0.001 ^a^	0.033 ± 0.002 ^b^
	Pentadecanoic acid C15:0	0.010 ± 0.000 ^a^	0.008 ± 0.000 ^b^
	Palmitic acid C16:0	0.580 ± 0.010 ^a^	0.449 ± 0.048 ^b^
	Heptadecanoic acid C17:0	0.019 ± 0.001 ^a^	0.017 ± 0.001 ^a^
	Stearic acid C18:0	0.313 ± 0.010 ^a^	0.249 ± 0.016 ^b^
	total	0.969 ± 0.022 ^a^	0.757 ± 0.068 ^b^
Monounsaturated fatty acid (MUFA)	Tetradecenoic acid C14:1	0.009 ± 0.000 ^a^	0.007 ± 0.001 ^b^
	Palmitoleic acid C16:1	0.053 ± 0.001 ^a^	0.039 ± 0.005 ^b^
	Heptadecenoic acid C17:1	ND	0.010 ± 0.001
	Trans-oleic acid C18:1n9t	ND	0.005 ± 0.001
	Oleic acid C18:1n9c	0.651 ± 0.142 ^a^	0.696 ± 0.074 ^a^
	total	0.714 ± 0.143 ^a^	0.757 ± 0.080 ^a^
Polyunsaturated fatty acid (PUFA)	Linoleic acid C18:2n6c	0.213 ± 0.017 ^a^	0.234 ± 0.011 ^a^
	Arachidonic acid C20:4n6	ND	0.042 ± 0.006
	total	0.213 ± 0.017 ^b^	0.276 ± 0.017 ^a^
Total fatty acids		1.896 ± 0.135 ^a^	1.789 ± 0.164 ^a^

Note: the different letters indicate significant difference (*p* < 0.05).

**Table 4:** Alpha-diversity index of bacteria in beef.

Sample	Chao1	Faith_pd	Goods_ Coverage	Observed_ Species	Pielou_e	Shannon	Simpson
Unfermented beef jerky	492.91	169.17	1.00	487.07	0.84	7.46	0.99
Fermented beef jerky	221.70	32.96	1.00	196.90	0.45	3.42	0.76

## Data Availability

The original contributions presented in the study are included in the article, further inquiries can be directed to the corresponding author.
